# Bio-Inspired Iron Pentadentate Complexes as Dioxygen Activators in the Oxidation of Cyclohexene and Limonene

**DOI:** 10.3390/molecules28052240

**Published:** 2023-02-28

**Authors:** Katarzyna Rydel-Ciszek, Tomasz Pacześniak, Paweł Chmielarz, Andrzej Sobkowiak

**Affiliations:** Department of Physical Chemistry, Faculty of Chemistry, Rzeszów University of Technology, Al. Powstańców Warszawy 6, 35-959 Rzeszów, Poland

**Keywords:** dioxygen activation, cyclohexene oxidation, limonene oxidation, iron-pentadentate complexes

## Abstract

The use of dioxygen as an oxidant in fine chemicals production is an emerging problem in chemistry for environmental and economical reasons. In acetonitrile, the [(N4Py)Fe^II^]^2+^ complex, [N4Py—*N,N*-bis(2-pyridylmethyl)-*N*-(bis-2-pyridylmethyl)amine] in the presence of the substrate activates dioxygen for the oxygenation of cyclohexene and limonene. Cyclohexane is oxidized mainly to 2-cyclohexen-1-one, and 2-cyclohexen-1-ol, cyclohexene oxide is formed in much smaller amounts. Limonene gives as the main products limonene oxide, carvone, and carveol. Perillaldehyde and perillyl alcohol are also present in the products but to a lesser extent. The investigated system is twice as efficient as the [(bpy)_2_Fe^II^]^2+^/O_2_/cyclohexene system and comparable to the [(bpy)_2_Mn^II^]^2+^/O_2_/limonene system. Using cyclic voltammetry, it has been shown that, when the catalyst, dioxgen, and substrate are present simultaneously in the reaction mixture, the iron(IV) oxo adduct [(N4Py)Fe^IV^=O]^2+^ is formed, which is the oxidative species. This observation is supported by DFT calculations.

## 1. Introduction

Although the oxygen molecule in its triplet ground state is diradical, contrary to expectations, it does not react in a way typical for radicals. It is unreactive with respect to the diamagnetic organic substrates. According to the Pauli exclusion principle, dioxygen to be able to react with diamagnetic substrates needs to change spin, but this process requires not only energy but also time, approximately 10^−9^ s. However, the lifetime of the active complex is considerably shorter, approximately 10^−13^ s, and the change of spin during its existence is impossible [1]. For this reason, dioxygen has to be activated to react with organic compounds. One route of dioxygen activation is its interactions with transition metal complexes. Knowledge of the dioxygen activation mechanism can contribute to understanding the reaction mechanisms that occur in living organisms (the action of enzymes, oxygen toxicity) [2]. Proteins containing non-heme iron centers are widespread in nature, for example, cytochrome P450 [3], Rieske oxygenases [4,5], or iron-activated bleomycin [6,7]. They exhibit a variety of activities, in particular, they activate dioxygen and hydroperoxides [8,9,10,11,12] mainly for selective oxidation of C-H bonds [5,13,14,15,16,17].

The possibility of dioxygen activation by metalloenzymes has inspired a vast number of papers, in which the interactions of transition metal complexes with dioxygen and hydroperoxides for the oxidation of organic compounds have been investigated. From the very beginning, a controversy has arisen about whether the oxidative species in such systems is a free hydroxyl radical or an oxygen adduct with the metal complex [18,19,20,21,22,23,24,25,26].

In recent years, the structures of oxygen adducts with transition metal complexes that possess pentadentate and tetradentate ligands containing nitrogen atoms (for example, in the case of iron *L*Fe^IV^=O, *L*Fe^III^-OOH, and *L*Fe^III^-OO-Fe^III^*L*) have been characterized in detail [10,16,27,28,29,30,31]. These species have been found to react with C-H bonds of various organic substrates [15,32,33,34,35,36,37,38]. The other group of metal dioxygen adducts shows the structures *L*Fe^V^=O and *L*Fe^V^=O(OH) [16,39,40]. These adducts, in addition to activating the C-H bond, cause also the oxidation of water [40].

The oxidative functionalization of hydrocarbons to useful chemicals using environmentally friendly oxidants under mild conditions is still a challenging area of chemistry [41,42,43,44]. Dioxygen (or air) is the best oxidant because of its low cost and significant environmental advantages. Both cyclohexene and limonene are essential industrial raw materials. Their catalytic oxidation can lead to the formation of useful synthetic intermediates used in fragrance, medicine, pesticide, surfactant, and polymer materials.

**Scheme 1:**
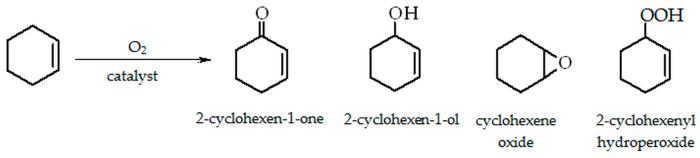
Products of cyclohexene oxidation.

Cyclohexene contains both C=C and C-H bonds, therefore, in the presence of dioxygen, it is susceptible to allylic oxidation [45]. Scheme 1 presents the products of the cyclohexene oxidation reaction: ketone (2-cyclohexen-1-one), alcohol (2-cyclohexen-1-ol), epoxide (cyclohexene oxide), and hydroperoxide (2-cyclohexenyl hydroperoxide).

Over the past three decades, the oxidation of cyclohexene by dioxygen has been performed under various conditions using homogeneous and heterogeneous catalysts in different solvents and in a solvent-free environment. A recent review summarizes the controllable and selective routes for the oxidation of cyclohexene [46].

There are not many studies using homogeneous catalysts in the process. It has been found that ruthenium(II) complexes with 2,2′-bipyridine (bpy), triphenylphosphine (Ph_3_P) [47], and with tris(2-pyridylmethyl)amine (TPA) [48] catalyze the oxidation of cyclohexene by dioxygen in acetonitrile to form mainly ketone and alcohol, epoxide was produced in much smaller quantities. In acetonitrile, the ruthenium(IV)-oxo complex [(py)(bpy)_2_Ru^IV^=O]^2+^ oxidizes cyclohexene to its ketone and alcohol [49,50]. As the ratio of cyclohexene to [*L*Ru^IV^=O]^2+^ increases, the ratio of ketone to alcohol decreases [50].

Schiff-base iron and manganese complexes have also been used as catalysts for cyclohexene oxidation with dioxygen. A manganese complex with salicylaldehyde and L-phenylalanine was reported to catalyze the discussed process; ketone, alcohol, and hydroperoxide were found as products [51]. Our research [52] has indicated that several manganese(III) complexes in combination with *tert*-butyl hydroperoxide activate dioxygen to oxygenate cyclohexene to its ketone, alcohol, and epoxide. The product profiles depend on the composition of the ligand and solvent. With picolinate, 2,2′-bipyridine, and triphenylphosphine oxide as ligands in pyridine/acetic acid (2:1 molar ratio) the dominant product is ketone, while Schiff–base complexes produce ketone, alcohol and epoxide in almost equal yields. However, in MeCN ketone is the dominant product for all of the complexes. We have also found [53] that the [(salen)Fe^III^]^+^ complex [salen—*N*,*N*′-ethylenebis(salicylimine)] is a useful catalyst for the cyclohexene oxidation with dioxygen in acetonitrile, ketone and alcohol are formed as main products.

Considering iron-based catalysts, we have reported [54] that labile iron(II) and iron(III) aqua or bpy complexes in acetonitrile activate dioxygen for the direct oxygenation of cyclohexene to produce mainly ketone and alcohol, epoxide was formed in much smaller amounts. It has been postulated that the oxidation process occurs within iron hydroperoxide complexes that also contain a substrate (Fe-OO-R). It is worth to notice that the formation of a similar complex has been proposed during the activation of dioxygen by the mononuclear cobalt(II) complex with tris [2-(*N*-tetramethylguanidyl) ethyl]amine in the presence of tetrahydrofuran and cyclohexene [55]. It has also been reported [34] that a non-heme iron(II) complex with TMC (1,4,8,11-tetramethyl-1,4,8,11- tetraazacyclotetradecane) activates dioxygen and generates its corresponding iron(IV)-oxo complex in the presence of substrates with weak C-H bonds (e.g., olefins and alkylaromatic compounds). Another study [35] has shown that an iron complex with tetraamido macrocyclic ligand [(TAML)Fe^III^]^−^ in acetonitrile is an efficient and selective catalyst for the oxidation of cyclohexene with dioxygen. For the low substrate-to-catalyst ratio only ketone and much smaller amounts of epoxide were detected, no alcohol was formed. It has been suggested that an iron(V)-oxo complex, [(TAML)Fe^V^(O)]^−^, formed in the presence of dioxgen and cyclohexene, was responsible for the initiation of the cyclohexene oxidation process.

**Scheme 2:**
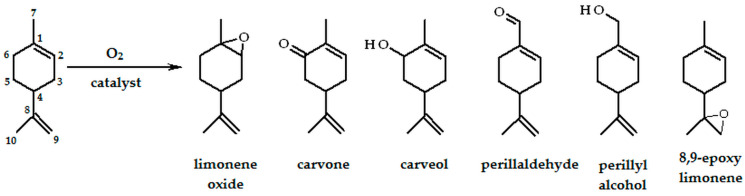
Possible products of limonene oxidation.

Limonene (1-isopropenyl-4-methylcyclohexene) is a cheap and abundant raw material; its oxidation products are high value compounds used mainly in the flavor and fragrance industry [56]. Therefore, the oxidation of limonene attracts attention, and a recent review [57] presents the state of the art of terpene oxidation processes. Possible products of limonene oxidation are presented in Scheme 2.

Using dioxygen and homogeneous catalysts, the following systems have been applied for limonene oxidation. The first is the Wacker oxidation process using PdCl_2_/CuCl_2_/O_2_ in glacial acetic acid [58,59,60,61]. Carvyl acetates, carveol, and carvone are among the major products. The other approach uses cobalt(II) salts as catalysts [62,63]. Co(OAc)_2_/Br^–^/O_2_ in acetic acid gives products similar to the Wacker system [62] while the CoCl_2_/O_2_ system in acetonitrile [63] gives three main products: limonene oxide (in acetic acid: mixture of limonene glycol and its acetates), carvone, and carveol. Aerobic oxidation of limonene under Mukaiyama conditions (atmospheric pressure of dioxygen, transition metal complex in the presence of an excess of sacrificial aldehyde) gives mainly 1,2-epoxylimonene [64,65,66], although small amounts of 8,9-epoxylimonene have also been observed. We have found that labile iron(II), iron(III) [67], and manganese(II) [68] complexes with 2,2′-bipyridine [Fe^II^(bpy)_2_]^2+^, [Fe^III^(bpy)_2_]^3+^, and [Mn^II^(bpy)_2_]^2+^ (prepared in situ by combination of the appropriate amounts of components) catalyze limonene oxidation by dioxygen in acetonitrile. Carvone, carveol, 1,2-epoxylimonene, and perillaldehyde were the main products. The iron(III) complex is reduced by the substrate to iron(II), which activates dioxygen. Perillaldehyde is likely formed directly from oxidation of the methyl group (not via alcohol). In the case of manganese(II), the reaction efficiencies after 24 h reaction time are approximately 5-times higher than those obtained for analogous iron(II) complexes, however, the 5 h long induction period was observed. When *t*-BuOOH was present in the reaction mixture, the induction period did not appear.

Taking into account the findings, we have decided to apply the iron(II) complex with the pentadentate ligand N4Py [*N*,*N*-bis(2-pyridylmethyl)-*N*-(bis-2-pyridylmethyl) amine] as the catalyst for the oxidation of cyclohexene and limonene by dioxygen in acetonitrile. The catalyst has not yet been used for aerobic oxidation of limonene. The complex [(N4Py)Fe^II^]^2+^ and its adduct with oxygen [(N4Py)Fe^IV^=O]^2+^ have been the subject of many investigations. The available literature provides detailed information on their structures derived from X-ray crystallography [69,70,71], UV-vis [71,72,73,74], NMR [70,71], EPR [73], and EXAFS [75] spectra, as well as electrospray ionization mass spectra [74], and Mössbauer parameters [9].

## 2. Results and Discussion

### 2.1. Oxidation of Cyclohexene

In acetonitrile dioxygen is activated by [(N4Py)Fe^II^]^2+^ for the oxidation of cyclohexene to form mainly ketone (2-cyclohexen-1-one) and alcohol (2-cyclohexen-1-ol). Epoxide is produced in much smaller amounts. Our previous study [54] indicated that under the same experimental conditions in the absence of a catalyst no cyclohexene oxidation was observed and in the presence of uncomplexed iron(II) only traces of ketone and alcohol were formed. In this study, it has been found that in a solution containing the substrate and catalyst in the oxygen-free atmosphere no oxidation products were found.

Table 1 presents the products concentrations after 24 h reaction time for dioxygen and air used as oxidants for different catalyst and substrate concentrations.

For example, as follows from the results shown in Table 1 after 24 h reaction time in dioxygen atmosphere, using 1 M cyclohexene and 1 mM catalyst, the products obtained were 114 mM ketone, 77 mM alcohol, and 10 mM epoxide, which means that approximately 0.2 M of the substrate has been reacted with the 201 product/catalyst turnovers. In the air atmosphere under the same experimental conditions, the product concentrations were slightly lower. The results presented also indicate that for 1 M cyclohexene, the increase in catalyst concentration from 0.5 to 2.5 mM in the case of dioxygen and to 5 mM in the case of air does not cause significant differences in the amounts of all products formed. For the catalyst concentration equal to 5 mM, a decrease in ketone concentration was observed when dioxygen was used and its increase in the case of air use. For higher catalyst concentrations (7.5 and 10 mM), no products were detected, which demonstrates that at higher concentrations the catalyst is decomposed. This behavior has been previously reported for iron-based catalysts that activate dioxygen [54,68]. For the catalyst concentration equal to 1 mM, an increase in cyclohexene concentration from 0.5 to 2 M causes almost a proportional increase in the amount of products both in the case of dioxygen and air. The presence in the reaction mixture of iodosobenzene (PhIO), which in the reaction with [(N4Py)Fe^II^]^2+^ gives [(N4Py)Fe^IV^=O]^2+^ [72], causes the increase of the concentration of the products formed, however the molar ratio of the products is approximately the same. The presence of small amounts of water in the reaction mixture causes a slight decrease in the yield of the products formed (Table S1).

**Figure 1:**
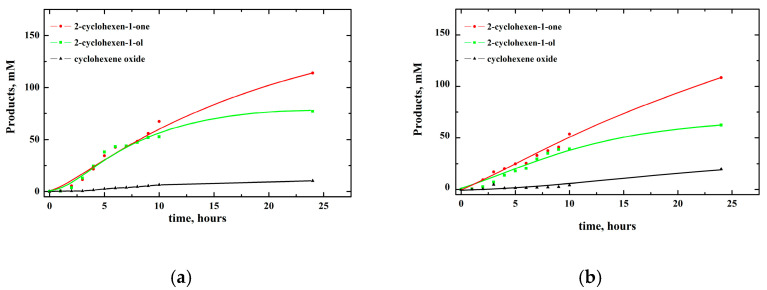
Dependence of products concentrations on time for oxidation of 1 M cyclohexene with (**a**) dioxygen (p_O2_ = 1 atm) and (**b**) air (p_O2_ = 0.2 atm) catalyzed by 1mM [(N4Py)Fe^II^]^2+^ in MeCN.

The dependence of product concentrations on time for oxidation with dioxygen and air is illustrated in Figure 1. The concentration profiles suggest that the products are formed independently.

### 2.2. Oxidation of Limonene

Similar to cyclohexene, limonene is oxidized in acetonitrile by dioxygen. The process catalyzed with [(N4Py)Fe^II^]^2+^, gives limonene oxide, carvone, and carveol as main products, and much smaller amounts of perillaldehyde and perillyl alcohol are also formed. As in the case of cyclohexene, appropriate blank experiments with limonene did not produce oxidation products or only traces when uncomplexed iron salt was used as catalyst [68].

The products concentrations obtained after 24 h reaction time for different catalyst and substrate concentrations using oxygen and air as oxidants are presented in Table 2. For example, the combination of 1 mM catalyst, 1 M limonene in dioxygen atmosphere after 24 h gave 89 mM limonene epoxide, 52 mM carvone, 31 mM carveol, 6 mM perillaldehyde, and 3 mM perillyl alcohol, which means that approximately 0.18 M of the substrate has reacted with the 181 product/catalyst turnovers. This indicates that the reaction efficiency is slightly lower in comparison to that of cyclohexene. As in the case of cyclohexene oxidation, the addition of iodosobenzene (PhIO) to the reaction mixture causes the concentration of the products formed to increase, and the molar ratio of the products is approximately the same.

As follows from the data presented in Table 2 for 1 M limonene, for low catalyst concentrations (0.5–1 mM), the use of dioxygen gave higher yields than the use of air, while the increase in its concentration (2.5–10 mM) caused the opposite effect to occur. It might suggest that the rate of the decomposition of the catalysts is higher in the case of the use of dioxygen, however in contrast to cyclohexene, for 7.5 and 10 mM of catalyst the products of limonene oxidation were formed. For all higher catalyst concentrations (5, 7.5, and 10 mM), the amounts of the products formed were similar for both dioxygen and air. It should be also noticed that for the catalyst concentration equal to 1 mM, there are no substantial differences among the yields of the products formed for different concentrations of limonene. As in the case of cyclohexene oxidation, the addition of small amounts of water to the reaction environment results in a slight decrease in the concentrations of the observed products (Table S2).

**Figure 2:**
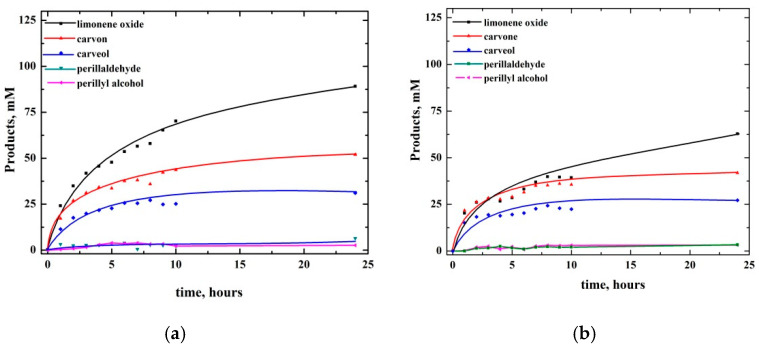
Dependence of products concentrations on time for oxidation of 1 M limonene with (**a**) dioxygen (p_O2_ = 1 atm), (**b**) air (p_O2_ = 0.2 atm) catalyzed by 1mM [(N4Py)Fe^II^]^2+^ in MeCN.

Figure 2 presents the dependence of the products concentrations on time. The product profiles suggest that, similar to cyclohexene, they are formed independently.

### 2.3. Electrochemical Investigation of the [(N4Py)Fe^II^]^2+^ Complex

**Figure 3:**
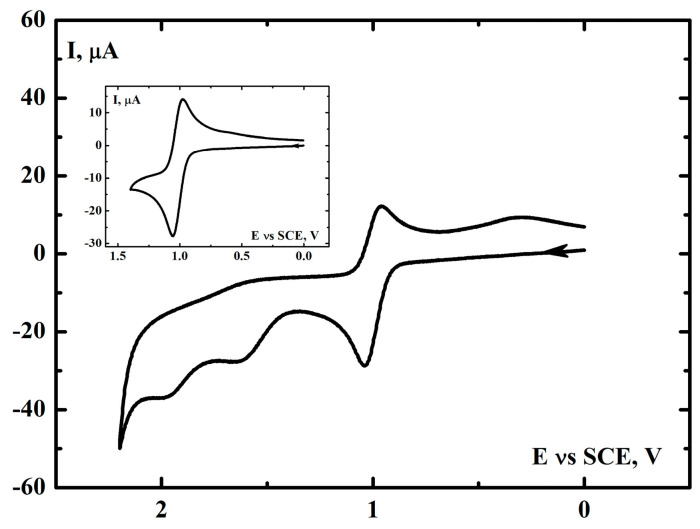
Cyclic voltammogram of 5mM [(N4Py)Fe^II^]^2+^in MeCN containing 0.1 M (*t*-Bu)_4_NClO_4_ as a supporting electrolyte in argon atmosphere. Scan rate, 0.1 V∙s^−1^, GCE (0.008 cm^2^), SCE vs. NHE +0.242 V. Inset, the anodic scan has been reversed after the appearance of the first peak.

Our previous research [54,67,68] indicated that cyclohexene and limonene interact with iron and manganese complexes used as catalysts for the oxygenation of these substrates. Electrochemical measurements can provide useful information in this area. Figure 3 presents the cyclic voltammogram of [(N4Py)Fe^II^]^2+^ in acetonitrile. Three anodic peaks have been observed at potentials equal to +1.05 V, +1.65 V, and +1.95 V (vs. SCE). The electrochemical behavior of [(N4Py)Fe^II^]^2+^ has previously been reported [71,76,77,78] however, it was limited to the first anodic peak (Figure 3 inset).

Electrochemical oxidation of [(N4Py)Fe^II^]^2+^ at +1.05 V is a reversible, diffusion-controlled process, which is confirmed by the linear dependence of the heights of the anodic and subsequent cathodic peaks on the square root of the scan rate (*ν*^1/2^) [79] (Figure S1 in the Supplementary Materials). There is a common agreement that at this potential oxidation of iron(II) to iron(III) takes place.

[(N4Py)Fe^II^]^2+^ -*e^−^* → [(N4Py)Fe^III^]^3+^ (1)

Since iron(III) is reduced by N4Py [69], the electrochemical oxidation of [(N4Py)Fe^II^]^2+^ can be used as a way to prepare [(N4Py)Fe^III^]^3+^.

The second oxidation peak at +1.65 V is irreversible but also diffusion controlled (the plot of its height vs. square root from scan rate is linear, Figure S2b). This behavior indicates that oxidation of iron(III) to iron(IV) probably occurs.

[(N4Py)Fe^III^]^3+^ -*e^−^* → [(N4Py)Fe^IV^]^4+^ (2)

This hypothesis is supported by the fact that high-valent transition metal ions can be generated by electrochemical oxidation in aprotic solvents [80,81,82].

The third irreversible oxidation peak that occurs at +1.95 V is not controlled by the diffusion process, rather by the adsorption/kinetic process, which is confirmed by the linear dependence of the peak height on the scan rate (*ν*) [79] (Figure S3), and also by the slight decrease in the dependence of the cathodic peak current at +1.0 V on (*ν*^1/2^) for higher scan rates (Figure S4). This suggests that [(N4Py)Fe^IV^]^4+^ reacts with basic electrolyte components, including traces of water, to form the iron(IV) oxo adduct [(N4Py)Fe^IV^=O]^2+^ and/or other iron oxygen adducts. Such a possibility is confirmed by the existence of the broad cathodic peak at +0.25 V observed in the reverse cathodic scan originated after the appearance of the anodic peaks at +1.65 V and +1.95 V (see Figure S1 and Figure S2a, respectively). The peak is not present when the anodic scan is reversed after the appearance of the anodic peak at +1.05 V. On the basis of the results presented in the following paragraph, the cathodic peak discussed can be attributed to the iron(IV) oxo species. The substantial difference between the oxidation potential of the iron(III) complex and the reduction potential of the iron(IV) oxo complex is probably caused by the presence of acetonitrile in coordination sphere of iron(III) and iron(II) complexes and its absence in coordination sphere of iron(IV) oxo complex. It has been shown [77,83] that the presence of acetonitrile in coordination sphere of an iron complex causes its redox potential to shift toward more positive values. Moreover, the generation of [(N4Py)Fe^IV^=O]^2+^ by the electrochemical oxidation of [(N4Py)Fe^II^]^2+^ in acetonitrile has been reported [76].

**Figure 4:**
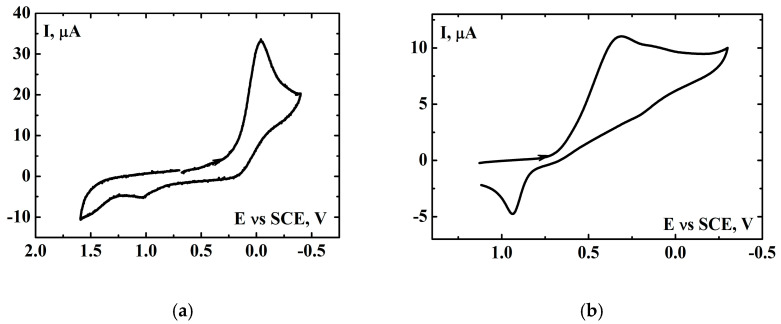
Cyclic voltammograms of (**a**) the mixture of 5mM [(N4Py)Fe^II^]^2+^ and 10 mM PhIO in MeCN [0.1 M (*t*-Bu)_4_NClO_4_] and (**b**) after the addition of 15 mM HClO_4_ to the solution shown in (**a**). Scan rate, 0.1 V∙s^−1^, GCE (0.008 cm^2^), SCE vs. NHE +0.242 V.

It is known that [(N4Py)Fe^IV^=O]^2+^is formed in a reaction of [(N4Py)Fe^II^]^2+^ with iodosobenzene (PhIO) [72]. Figure 4a presents a voltamogram registered in the solution containing [(N4Py)Fe^II^]^2+^ and PhIO, and the cathodic peak present at 0.0 V results in a reduction of [(N4Py)Fe^IV^=O]^2+^ [71,76,77,78]. Iodosobenzene alone does not exhibit any electrochemical activity in the investigated potential region (Figure S5). The presence of a proton source in the solution mentioned above causes the iron(IV) oxo reduction peak to shift toward more positive values, approximately +0.3 V. In the both cases, in the reversed scan a small anodic peak was observed around +1.0 V, which corresponds to the electrooxidation of [(N4Py)Fe^II^]^2+^.

**Figure 5:**
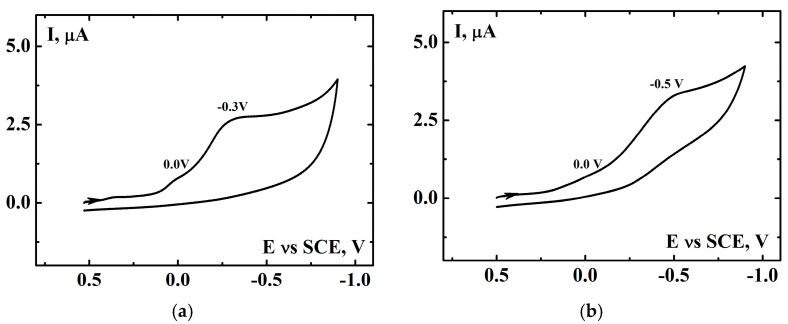
Cyclic voltammograms for 1 mM [(N4Py)Fe^II^]^2+^ in air atmosphere after 5 h of introduction of (**a**) 1 M cyclohexene and (**b**) 1 M limonene into the system, registered in MeCN [0.1 M (*t*-Bu)_4_NClO_4_]. Scan rate, 0.1 V∙s^−1^, GCE (0.008cm^2^), SCE vs. NHE +0.242 V.

Voltammetric measurements were used to check for possible interactions between the catalyst and the investigated substrates. A catalyst concentration equal to 1 mM has been applied to mimic the oxidation processes performed (cyclohexene is not oxidized when the catalyst concentration exceeds 5). Figure 5 presents voltamograms of a combination of 1 mM catalyst, 1 M cyclohexene (Figure 5a), or limonene (Figure 5b) after 5 h in air atmosphere. In the both cases, a small reduction peak was observed at 0.0 V, which corresponds to a reduction of [(N4Py)Fe^IV^=O]^2+^.

This indicates that the iron(IV) oxo species is formed when the catalyst, substrate, and dioxygen are present simultaneously in the reaction mixture. The other reduction peaks visible in the voltammograms at more negative potentials (–0.3 V and –0.5 V for cyclohexene and limonene, respectively) are probably caused by the reduction of the other iron-oxygen species, which can be formed in the reaction mixture. Figures S6 and S7 show a series of voltammograms, presented separately for the anodic or cathodic scan recorded first, which have led to the results presented in Figure 5. It is worth to notice that the voltammograms confirm that [(N4Py)Fe^II^]^2+^ is air stable in acetonitrile [77]. The presence of dioxygen in the solution (without organic substrate) has a minimal effect on the potential and height of the first oxidation peak (compare curves II and III in Figure S6a). Additionally, any reduction peak is present in the first cathodic scan (Figures S6b and S7b, curve III), which means that iron(III) is not formed in the presence of dioxygen.

### 2.4. DFT Calculations

**Figure 6:**
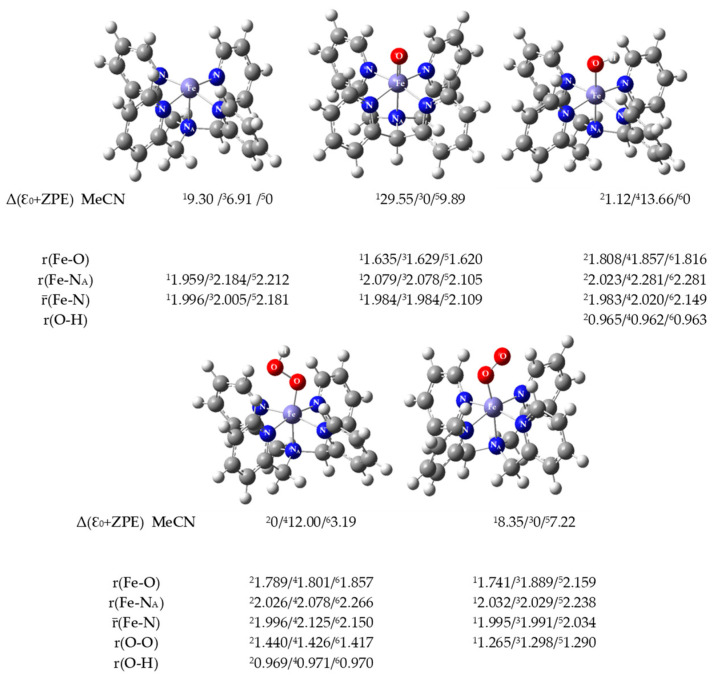
Optimized structures, visualized with GaussView, of iron complexes with relative electronic energies (in kcal/mol). The zero energy level corresponds to the most stable structure in the multiplicity examined. The key bond lengths (in Å) are presented for ^1^-singlet/^3^-triplet/^5^-quintet or ^2^-doublet/^4^-quarted/^6^-sextet states, respectively. Def2TZVP method with MeCN as the PCM model.

In order to better understand the mechanism of reactions catalyzed by the [(N4Py)Fe^II^]^2+^/O_2_ system, in addition to preparative and electrochemical methods, quantum calculations were also conducted. DFT calculations were performed for [(N4Py)Fe^II^]^2+^ and for the possible structures that the complex can form with the oxygen molecule, and the optimized structures are shown in Figure 6. The calculations were made for various spin states of the complex molecules using MeCN as the solvent model and the results of the calculated thermodynamic parameters (energies, enthalpies, and free energies) are shown in Table S3.

**Figure 7:**
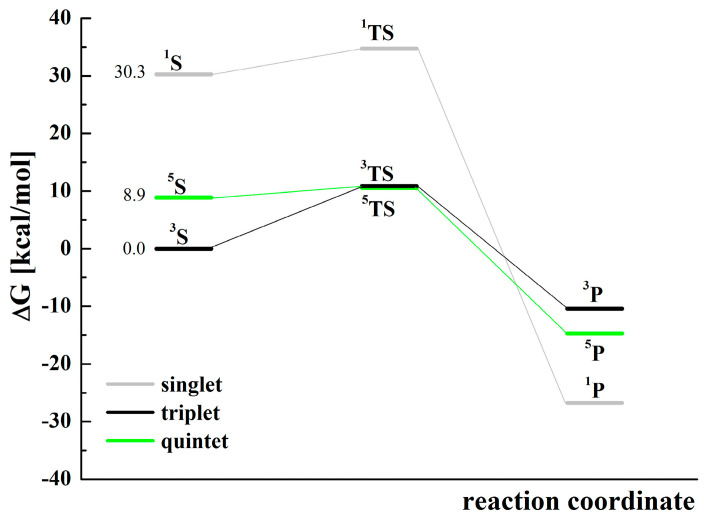
Relative Gibbs free energies (Table S5) of the singlet (1, gray), triplet (3, black), and quintet (5, green) states for the reaction of cyclohexene oxidation by [(N4Py)Fe^IV^=O]^2+^ with the use of MeCN as a solvent model. For the substrates S (in three various spin states), the values of starting relative Gibbs free energies are given next to the corresponding levels. The symbols used: S—substrates: [(N4Py)Fe^IV^=O]^2+^ + H-C_6_H_9_, TS—transition state: [(N4Py)Fe---O---H-C_6_H_9_]^2+^, and P—products: [(N4Py)Fe^III^-OH]^2+^ + C_6_H_9_.

Based on the data presented in Table S3, the Gibbs free energies corresponding to the reactions shown in Table S4 were calculated for the basic optimized structures, visualized in Figure 6. Table S4 shows that under standard conditions, most catalyst activation reactions proceed spontaneously (***∆****_r_G* < 0). Electrochemical studies have shown that the presence of an organic substrate, as a hydrogen donor, is necessary for the formation of reactive iron(IV) oxo species. In DFT studies of cyclohexene oxidation with dioxygen, transformations of oxygen adducts such as: [(N4Py)Fe^IV^=O]^2+^ (Figure 7 and Figure S8, Tables S5 and S6) and the hypothetical one [(N4Py)Fe^III^OOC_6_H_9_]^2+^ (Figure S9, Table S7) were analyzed. The reaction profile of the hydrogen atom transfer from cyclohexene to ^1,3,5^[(N4Py)Fe^IV^=O]^2+^ is presented in Figure 7, and related thermodynamic data are collected in Table S5. Substrates (S) [(N4Py)Fe^IV^=O]^2+^ + C_6_H_9_-H, through the transition state (TS), give products (P) [(N4Py)Fe^III^-OH]^2+^ + C_6_H_9_ according to reaction:

[(N4Py)Fe^IV^=O]^2+^ + H-C_6_H_9_ ↔ [(N4Py)Fe^III^---O---H-C_6_H_9_]^2+^ → [(N4Py)Fe^III^-OH]^2+^ + C_6_H_9_ (3)

The activation energies (*E*_a_) of the reaction (3) for the analyzed states: singlet, triplet, and quintet, calculated on the basis of relative Gibbs free energies, are equal to 4.5, 10.9, and 1.7 kcal/mol, respectively (Figure 7 and Table S5), whereas the values calculated using relative electronic energies are equal to 2.5, 8.3, and 0.1 kcal/mol. The values change in the same order. The data indicate that the quintet state is the most favorable because it has the lowest activation energy. However, due to the lowest relative energies of the substrates in the triplet state, the state is also probable. The occurrence of substrates in the singlet state, because of the significant difference in energy about 30 and 21 kcal/mol higher compared to the triplet and quintet states, respectively, is unexpected.

High-valent iron(IV) oxo complexes can react with alcohols giving iron(III) hydroxy species [84], therefore the thermodynamic parameters for the reaction of [(N4Py)Fe^IV^=O]^2+^ with C_6_H_9_OH were also calculated. The reaction profile is presented in Figure S8, and the calculated data are collected in Table S6. For this reaction, the triplet and quintet states were analyzed only because substrates in the singlet multiplicity had energy 33 and 25 kcal/mol higher than the triplet and quintet states, respectively (Table S6). For the reaction of alcohol oxidations, the triplet state of [(N4Py)Fe^IV^=O]^2+^ is privileged, however the activation energy of hydrogen atom transfer from the substrate to the iron(IV) oxo species is higher than that for cyclohexene. This result supports the experimental finding that 2-cyclohexen-1-ol is not oxidized in the presence of cyclohexene excess. The parameters calculated for the transformation of hypothetical adduct [(N4Py)Fe^III^OOC_6_H_9_]^2+^ toward the formation of the ketone (Figure S9, Table S7) have shown that the process has a high activation energy (127 kcal/mol was the lowest value).

In the cyclohexene oxidation process, the [(N4Py)Fe^IV^=O]^2+^ adduct seems to be the most probable reactive species. Therefore, only this adduct was used to model the limonene oxidation reaction. Based on the data collected in Table S8 and presented in Figure S10, it was found that the activation energies (calculated on the basis of relative Gibbs free energies) for the hydrogen atom transfer from limonene to the complex [(N4Py)Fe^IV^=O]^2+^ are higher than for cyclohexene oxidation and are equal to 33.0 kcal/mol for the singlet, 25.9 kcal/mol for the triplet, and 40.5 kcal/mol for the quintet state. This indicates that the triplet state is the most favorable one.

The activation energy of hydrogen atom transfer from the cyclohexene to the iron(IV) oxo species is lower than that for limonene, which indicates that in the presence of this complex, the cyclohexene oxidation reaction should proceed more effectively than the limonene oxidation reaction. This observation is consistent with the catalytic results obtained for these two examined organic substrates. When comparing the data presented in Table 1 and Table 2, it can be seen that for the cyclohexene oxidation reaction, a significantly (even twice) higher number of TONs was obtained than for the reaction with limonene.

### 2.5. Considerations on Oxidation Mechanism

**Scheme 3:**
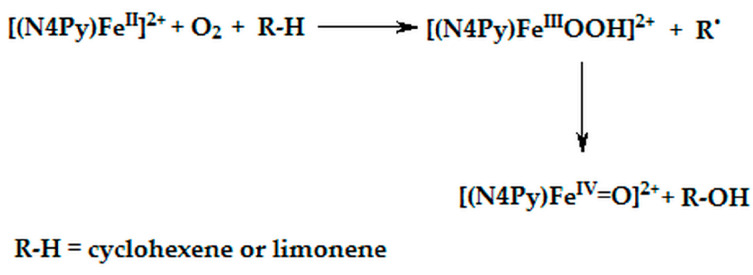
Proposed route for iron(IV) oxo species formation.

The experimental data presented indicate that [(N4Py)Fe^IV^=O]^2+^ adduct is the reactive species in the oxidation of cyclohexene and limonene. The adduct is formed when the catalyst [(N4Py)Fe^II^]^2+^, dioxygen and the substrate are simultaneously present in the reaction environment. The sequence of possible processes, which are presented in Scheme 3, leading to the formation of iron(IV) oxo species causes also the formation of an alcohol [30,34,77].

The assumption that [(N4Py)Fe^IV^=O]^2+^ is the reactive intermediate allows easy explanation of the formation of an alcohol and epoxide by insertion of an oxygen atom into the substrate molecule. Equations (4) and (5) present the overall processes.

[(N4Py)Fe^IV^=O]^2+^ + R-H → [(N4Py)Fe^II^]^2+^ + R-OH (4)

[(N4Py)Fe^IV^=O]^2+^ + R-H → [(N4Py)Fe^II^]^2+^ + (O)R-H *(epoxide)* (5)

The mechanistic details of these transformations have been described in [85,86]. In the above processes [(N4Py)Fe^II^]^2+^ appears among the products, allowing the catalytic process to occur.

**Scheme 4:**
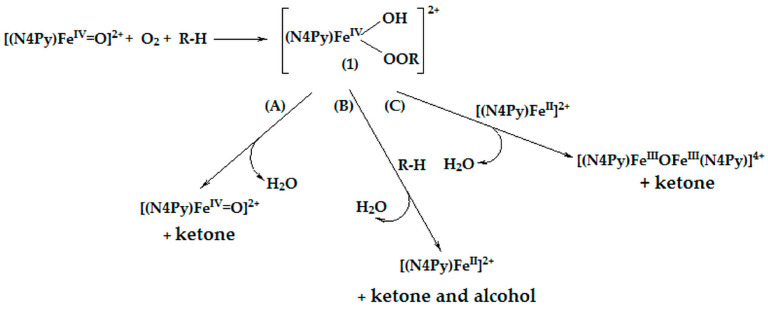
Tentative mechanism of the oxidation process leading to ketone formation.

However, to explain the formation of ketone during the oxidation process, this straightforward mechanism including the iron(IV) oxo intermediate cannot be applied. Taking into account our previous research [52,54,67,68] we propose that, in the reaction mixture, the combination of iron(IV) oxo species, dioxygen, and substrate produces a hypothetical adduct (**1**) (Scheme 4).

Possible transformations of the hypothetical adduct can include its: decomposition to form ketone and iron(IV) oxo species (path A), reaction with the substrate molecule to produce ketone and alcohol and also the catalyst (path B), and interaction with the catalyst that gives ketone and iron(III) μ-oxo complex, which in many cases is catalytically inactive (path C) [87,88].

In our previous research [54,68] structures analogous to the (**1**) were postulated as an intermediate step of the oxidation process when the Fe or Mn catalysts applied were coordinately unsaturated, e.g., they were prepared in-situ with fewer ligands then available metal coordination sites. The same type of structures was proposed for iron complexes with tetradentate ligands [16]. Since N4Py is a pentadentate ligand, the postulated mechanism seems to be questionable.

However, it has been shown [71] that in water solution of the [(N4Py)Fe^II^]^2+^ complex, one nitrogen atom of the N4Py ligand can free iron ion coordination site for water molecule or hydroxyl ion. By analogy, it can be assumed that such release of iron coordination place can be possible during the oxidation process investigated. It is necessary to emphasize that the proposed mechanism is a hypothetical one, but it gives reasonable explanation of the observed experimental data.

## 3. Materials and Methods

### 3.1. Equipment

The reaction products were separated and identified with a Hewlett-Packard 4890A Series gas chromatograph equipped with an HP-1 capillary column (cross-linked methyl-silicone gum phase, 30 m x 0.53 mm i.d). ^1^H and ^13^C NMR analysis was carried out in DMSO-d_6_ or CDCl_3_ using Bruker Avance 500 MHz spectrometer (Bruker, Karlsruhe, Germany) at 25 °C. Princeton Applied Research Model 273A potentiostat was used to perform voltammetric measurements.

### 3.2. Chemicals and Regents

The reagents used for the investigations and syntheses were of the highest purity commercially available and were used without further purification. The solvent for all experiments was acetonitrile (≥99.9%, HPLC grade) purchased from Aldrich. High-purity argon (grade 5.0) supplied by Linde (Poland) was used to de-aerate the solutions. Iron(II) perchlorate, Fe(ClO_4_)_2_·6H_2_O, sodium hydroxide (98%), biphenyl (PhPh, ≥99%), cyclohexene (≥99%), 2-cyclohexen-1-one (95%), 2-cyclohexen-1-ol (95%), cyclohexene oxide (98%), (R)-(+)-limonene (97%), (-)-carveol, mixture of isomers, (97%), (R)-(-)-carvone (98%), (+)-limonene oxide, mixture of *cis* and *trans*, (97%), (S)-(-)-perillyl alcohol (96%), (S)-(-)-perillaldehyde (92%), di-2-pyridilketone oxime (98%), 2-picolyl chloride hydrochloride (98%), iodobenzene (98%), and chloroform (98%) were obtained from Aldrich. Peracetic acid (39%) and perchloric acid (70%) were delivered by Fluka. Ammonium acetate (97%), sodium sulfate anhydrous (99%), magnesium sulfate anhydrous (99%), and ammonia solution (25%) were purchased from POCH (Poland). PhIO was synthesized by a well-known procedure [89,90].

### 3.3. Synthesis of [(N4Py)Fe^II^]^2+^

N4Py used as a ligand and its iron complex [(N4Py)Fe^II^]^2+^ were synthesized according to the procedure given in the literature [69,91,92] using the Schlenk line system. ^1^H NMR analysis with the use of DMSO-d_6_ or CDCl_3_ for the synthesized ligand; N4Py and the [(N4Py)Fe^II^]^2+^ complex confirmed that the characteristic bands [69] were obtained. ^1^H NMR N4Py (500 MHz, DMSO-d_6_, 25 °C): δ (ppm) = 3.86 (s, 4H, CH), 5.25 (s, 1H, CH), 7.22 (m, 4H, Py), 7.42 (m, 8H, Py), 8.48 (d, 2H, Py), 8.50 (d, 2H, Py). The obtained spectrum contained four doublets with chemical shifts in the range of 7.42–7.67 ppm derived from the hydrogen of the pyridine group connected to the ligand’s methine group. However, the chemical shift of the methine group had a slightly higher value than that reported in [69]. ^1^H NMR N4Py (500 MHz, CDCl_3_, 25 °C): δ (ppm) = 3.87 (s, 4H, CH), 5.23 (s, 1H, CH), 7.11 (m, 4H, Py), 7.63 (m, 8H, Py), 8.48 (d, 2H, Py), 8.63 (d, 2H, Py). ^13^C NMR analysis C (N4Py): δ (ppm) = 64.71 (CH_2_, aliphatic), 68.73 (CH_2_, aliphatic), 71.60 (CH, aliphatic), 122.9–149.0 ppm (CH, 2-pyridine), 159.85–162.36 (C, 2-pyridine).

### 3.4. Methods

Voltammetric measurements were performed in a 2 mL electrochemical cell with provision to control the presence of oxygen with an argon purge system. The working electrode was a 1 mm diameter glassy carbon in PEEK (Cypress Systems, Division of ESA, Inc., Wan Chai, Hong Kong), the auxiliary electrode—a platinum wire, and the reference electrode—Ag/AgCl wire in an aqueous tetramethylammonium chloride solution that was adjusted to give a potential of 0.00 V vs. SCE. The reference electrode was contained in a Pyrex tube with a Vycor tip, which was placed inside a Luggin capillary [93]. Before each experiment, the working electrode was polished using a Buehler Micropolish Alumina Gamma 3B and Buehler Microcloth polishing cloth, then rinsed with deionized water and dried.

During the oxidation processes, the appropriate amount of catalyst was dissolved in de-aerated acetonitrile (O_2_, 0 atm) followed by the addition of the substrate (usually 1 M, total volume equal to 2.5 mL) in the reaction cell (25 cm^3^ vial with cut-out cap and Teflon-faced septum). The excess of the substrate was used to minimize the over-oxidation of the initial oxidation products and protect the catalyst from oxidative degradation. The solution was saturated with dioxygen (O_2_, 1 atm) or air (O_2_, 0.2 atm). After saturation, dioxygen or air was passed over the solution during the duration of the experiment to maintain a constant concentration of dioxygen in the reaction mixture. The reactions were allowed to proceed for 24 h with constant stirring at room temperature (23 ± 1 °C). The samples (0.2 µL) were periodically withdrawn to follow the progress of the reaction, using gas chromatography (GC) analysis. Biphenyl (5 mM for cyclohexene oxidation or 10 mM for limonene oxidation) was used as an internal standard. The presented values of the products concentrations are the mean values of 3 independent experiments.

Calculations of thermodynamic parameters were made in Gaussian 16 [94] using DFT methods with the hybrid density functional B3LYP and the basis set Def2-SVP. The GaussView program was used to model the structures of both cyclohexene and limonene molecules as well as the different spin states of the [(N4Py)Fe^II^]^2+^ of complexes and their adducts with oxygen. Geometry optimization was performed using the B3LYP/Def2SVP method with the added D3 version of Grimme’s dispersion with Becke-Johnson damping [95,96]. The final electronic energies of the stationary points were calculated using the Def2-TZVP basis set with the added D3 version of Grimme’s dispersion with Becke-Johnson damping and the PCM model of acetonitrile. Reported in this paper values combine these electronic energies with the ZPE correction or Gibbs free energy correction. The most stable form of the calculated structures was used to calculate the Gibbs free energy for the reactions analyzed.

## 4. Conclusions

In spite of the fact that [(N4Py)Fe^II^]^2+^ complex does not react with dioxygen in acetonitrile their combination with an alkene causes its oxidation. The system is not selective, in the case of cyclohexene as the main products 2-cyclohexen-1-one and 2-cyclohexen-1-ol are formed roughly in the 2:1 molar ratio, whereas in the case of limonene 1,2-epoxylimonene, carvone, and carveol in the nearly 2:1.5:1 molar ratio occur after reaction. However, the system is twice as efficient as the [(bpy)_2_Fe^II^]^2+^/O_2_/cyclohexene system [54] and comparable to the [(bpy)_2_Mn^II^]^2+^/O_2_/limonene system [68].

The electrochemical measurements presented indicate that the [(N4Py)Fe^IV^=O]^2+^ adduct is formed during the interaction of the catalyst, dioxygen, and substrate in the cases of both cyclohexene and limonene. The adduct also is likely to form when the oxidation of [(N4Py)Fe^II^]^2+^ (without the presence of organic substrates) takes place at the potentials more positive than +1.65 V, i.e., the formation of the second oxidation peak. The discussed iron(IV) oxo adduct is considered as a reactive intermediate in the oxidation process [30,34]. Indeed the addition of PhIO to the reaction mixture, which causes the formation of [(N4Py)Fe^IV^=O]^2+^ [72] increases the amount of products formed both in the case of cyclohexene and limonene. The DFT calculations confirms that the adduct is a favorable oxidant.

## Figures and Tables

**Table 1:** Oxidation of cyclohexene with dioxygen (p_O2_ = 1 atm) and air (p_O2_ = 0.2 atm) catalyzed by [(N4Py)Fe^II^]^2+^ in MeCN. Reaction time 24 h.

Catalyst, mM	Substrate, M	O_2_, atm	Ketone, mM	Alcohol, mM	Epoxide, mM	TON
0.5	1	1	135	71	13	438
0.5	1	0.2	92	47	11	300
1	1	1	114	77	10	201
1	1	0.2	108	62	20	190
1 ^a^	1	0.2	144	74	23	241
2.5	1	1	115	28	12	62
2.5	1	0.2	104	55	8	67
5	1	1	51	32	3	17
5	1	0.2	126	50	7	37
1	0.5	1	55	18	9	82
1	0.5	0.2	39	10	8	57
*1* ^b^	*1*	*1*	*114*	*77*	*10*	*201*
*1* ^b^	*1*	*0.2*	*108*	*62*	*20*	*190*
1	1.5	1	174	107	15	296
1	1.5	0.2	177	100	16	293
1	2	1	248	119	42	409
1	2	0.2	293	129	40	462

TON—product molecules per catalyst molecule. ^a^—3 mM PhIO has been added to the reaction mixture. ^b^—repeated values.

**Table 2:** Oxidation of limonene with dioxygen (p_O2_ = 1 atm) and air (p_O2_ = 0.2 atm) catalyzed by [(N4Py)Fe^II^]^2+^ in MeCN. Reaction time 24 h.

Catalyst, mM	Substrate, M	Oxidant	Limonene Oxide, mM	Carvone, mM	Carveol, mM	Perill Aldehyde, mM	Perillyl Alcohol, mM	TON
0.5	1	1	48	33	24	0	0	210
0.5	1	0.2	43	32	22	1	3	202
1	1	1	89	52	31	6	3	181
1	1	0.2	63	42	27	3	3	138
1 ^a^	1	0.2	93	55	32	3	7	190
2.5	1	1	56	40	28	1	2	51
2.5	1	0.2	74	47	29	2	5	63
5	1	1	49	36	26	1	1	23
5	1	0.2	59	43	29	1	2	27
7.5	1	1	51	37	28	0	0	15
7.5	1	0.2	60	42	31	2	4	19
10	1	1	57	41	33	5	1	14
10	1	0.2	57	41	28	2	3	13
1	0.5	1	46	22	10	2	3	83
1	0.5	0.2	51	22	13	2	3	91
*1* ^b^	*1*	*1*	*89*	*52*	*31*	*6*	*3*	*181*
*1* ^b^	*1*	*0.2*	*63*	*42*	*27*	*3*	*3*	*138*
1	1.5	1	75	59	35	2	5	176
1	1.5	0.2	58	46	30	0	0	134
1	2	1	91	70	42	3	4	210
1	2	0.2	64	58	35	1	2	160

TON—product molecules per catalyst molecule. ^a^—3 mM PhIO has been added to the reaction mixture. ^b^—repeated values.

## Data Availability

The data presented in this study are available in the Supplementary Materials or on request from the corresponding author.

## Supplementary Materials

The following supporting information can be downloaded at: https://www.mdpi.com/article/10.3390/molecules28052240/s1, Table S1: Oxidation of 1 M cyclohexene with air (p_O2_ = 0.2 atm) catalyzed by 1 mM [(N4Py)Fe^II^]^2+^, in MeCN. Analysis of the effect of water addition. Reaction time 24 h; Table S2: Oxidation of 1M limonene with air (p_O2_ = 0.2 atm) catalyzed by 1 mM [(N4Py)Fe^II^]^2+^, in MeCN. Analysis of the effect of water addition. Reaction time 24 h; Figure S1: The dependence of the current on the square root of the scan rate (ν^1/2^) registered for 5 mM [(N4Py)Fe^II^]^2+^ in MeCN with 0.1 M (*t*-Bu)_4_NClO_4_ on a glassy carbon electrode (GCE 0.008 cm^2^), for: (a) the anodic peak at the potential +1.05 V, (b) the subsequent cathodic peak; Figure S2: The electrochemical behaviour of 5 mM [(N4Py)Fe^II^]^2+^ in MeCN with 0.1 M (*t*-Bu)_4_NClO_4_, GCE (0.008 cm^2^), SCE vs. NHE +0.242 V. (a) Cyclic voltammogram with anodic scan reversed after the appearance of the peak at potential +1.65 V, scan rate 0.1 V s^−1^. (b) The dependence of the current on the square root of the scan rate (ν^1/2^) for the anodic peak at potential +1.65 V; Figure S3: The dependence of the current registered for the anodic peak at +1.95 V on: (a) the square root of the scan rate (ν^1/2^) and (b) the scan rate, (ν), registered for 5 mM [(N4Py)Fe^II^]^2+^ in MeCN with 0.1 M (*t*-Bu)_4_NClO_4_ on a glassy carbon electrode (GCE 0.008 cm^2^); Figure S4: The dependence of the current of the cathodic peak at +1.0 V on the square root of the scan rate (ν^1/2^) registered for 5 mM [(N4Py)Fe^II^]^2+^ when the scan was reversed after the appearance of the anodic peak at +1.95 V, in MeCN with 0.1 M (*t*-Bu)_4_NClO_4_ on a glassy carbon electrode (GCE 0.008 cm^2^); Figure S5: Cyclic voltammogram of 15 mM PhIO in MeCN with 0.1 M (*t*-Bu)_4_NClO_4_, GCE (0.008 cm^2^), SCE vs. NHE +0.242 V; Figure S6: Cyclic voltammograms for 1 mM [(N4Py)Fe^II^]^2+^ in MeCN with 0.1 M (*t*-Bu)_4_NClO_4_, (a) the anodic scan, (b) the cathodic scan, was recorded first. I— basic electrolyte in Ar atmosphere, II—after addition of 1 mM [(N4Py)Fe^II^]^2+^ in Ar atmosphere, III—as (II) in air atmosphere, IV—as (III) after addition 1 M cyclohexene, V—as (IV) after 5 h. Scan rate, 0.1 V∙s^−1^, GCE (0.008 cm^2^), SCE vs. NHE +0.242 V; Figure S7: Cyclic voltammograms for 1 mM [(N4Py)Fe^II^]^2+^ in MeCN with 0.1 M (*t*-Bu)_4_NClO_4_, (a) the anodic scan, (b) the cathodic scan, was recorded first. I—basic electrolyte in Ar atmosphere, II—after addition of 1 mM [(N4Py)Fe^II^]^2+^ in Ar atmosphere, III—as (II) in air atmosphere, IV—as (III) after addition 1 M limonene, V—as (IV) after 5 h. Scan rate, 0.1 V∙s^−1^, GCE (0.008 cm^2^), SCE vs. NHE +0.242 V; Table S3: The energies (with and without zero point correction), enthalpies, free energies (G), and respective relative values for different catalyst molecules calculated with Def2TZVP and acetonitrile as PCM model; Table S4: Reaction Gibbs free energy for the activation of complexes; Table S5: The energies (with and without zero point correction), enthalpies, free energies (G), and respective relative values for the singlet (1), triplet (3), and quintet (5) states for reaction of cyclohexene oxidation catalyzed by [(N4Py)Fe^IV^=O]^2+^ in PCM model; Figure S8: Relative Gibbs free energies (Table S6) of the triplet (3, black) and quintet (5, green) states for the reaction of 2-cyclohexen-1-ol oxidation by [(N4Py)Fe^IV^=O]^2+^ with the use of MeCN as a solvent model. For the substrates S (in two various spin states), the values of starting relative Gibbs free energies are given next to the corresponding levels. The symbols used: S—substrates: [(N4Py)Fe^IV^=O]^2+^ + HO-C_6_H_9_, TS—transition state: [(N4Py)Fe---O---HO-C_6_H_9_]^2+^, P—products: [(N4Py)Fe^III^-OH]^2+^ + O-C_6_H_9_; Table S6: The energies (with and without zero point correction), enthalpies, free energies (G), and respective relative values for the singlet (1), triplet (3), and quintet (5) states for reaction of 2-cyclohexen-1-ol oxidation by [(N4Py)Fe^IV^=O]^2+^ in MeCN as PCM model; Figure S9: Relative Gibbs free energies (Table S7) of the doublet (2, gray), quartet (4, black), and sextet (6, green) states for transformations of [(N4Py)Fe^III^OOC_6_H_9_]^2+^ to [(N4Py)Fe^III^OH]^2+^ and ketone with the use of MeCN as a solvent model. For the substrates S (in three various spin states), the values of starting relative Gibbs free energies are given next to the corresponding levels. The symbols used: S—substrate: [(N4Py)Fe^III^OOC_6_H_9_]^2+^, TS—transition state: [(N4Py)Fe^III^O---OC_6_H_8_---H]^2+^, P—products: [(N4Py)Fe^III^OH]^2+^ + C_6_H_8_O; Table S7: The energies (with and without zero point correction), enthalpies, free energies (G), and respective relative values for the doublet (2), quartet (4), and sextet (6) states for the reaction of [(N4Py)Fe^III^OOC_6_H_9_]^2+^ (S) to [(N4Py)Fe^III^OH]^2+^ and ketone (P) in PCM model; Table S8: The energies (with and without zero point correction), enthalpies, free energies (G), and respective relative values for the singlet (1), triplet (3), and quintet (5) states for reaction of limonene oxidation by [(N4Py)Fe^IV^=O]^2+^ in PCM model; Figure S10: Relative Gibbs free energies (Table S8) of the singlet (1, gray), triplet (3, black), and quintet (5, green) states for the reaction of limonene oxidation by [(N4Py)Fe^IV^=O]^2+^ with the use of MeCN as a solvent model. For the substrates S (in three various spin states), the values of starting relative Gibbs free energies are given next to the corresponding levels. The symbols used: S—substrates: [(N4Py)Fe^IV^=O]^2+^ + H-C_10_H_15_, TS—transition state: [(N4Py)Fe---O---H-C_10_H_15_]^2+^, P—products: [(N4Py)Fe^III^-OH]^2+^ + C_10_H_15_.

## Sample Availability

Samples of the compounds are available from the authors.
