# Supplementary material for: Bio-Inspired Iron Pentadentate Complexes as Dioxygen Activators in the Oxidation of Cyclohexene and Limonene
Source: Molecules. 2023 Feb 28;28(5):2240. doi: 10.3390/molecules28052240 (PMC10004738; doi:10.3390/molecules28052240)
Supplement: Supplementary file 1 [file molecules-28-02240-s001.zip › molecules-2229425-supplementary.pdf]

Supplementary Materials

Bio-Inspired Iron Pentadentate Complexes as Dioxygen Activators in the Oxidation of Cyclohexene and Limonene

Katarzyna Rydel-Ciszek *, Tomasz Pacześniak, Paweł Chmielarz and Andrzej Sobkowiak *

Department of Physical Chemistry, Faculty of Chemistry, Rzeszów University of Technology, Al. Powstańców Warszawy 6, 35-959 Rzeszów, Poland
* Correspondence: kasiar@prz.edu.pl (K.R.-C.); asobkow@prz.edu.pl (A.S.)

Table S1. Oxidation of 1 M cyclohexene with air (p_{O2} = 0.2 atm) catalyzed by 1 mM $[(N4Py)Fe^{II}]^{2+}$, in MeCN. Analysis of the effect of water addition. Reaction time 24 h.

Water, mM	Ketone, mM	Alcohol, mM	Epoxide, mM	TON
0	108	62	20	190
10	32	45	2	79
50	41	45	3	89
100	45	42	3	90

TON - product molecules per catalyst molecule

Table S2. Oxidation of 1M limonene with air (p_{O2} = 0.2 atm) catalyzed by 1 mM $[(N4Py)Fe^{II}]^{2+}$, in MeCN. Analysis of the effect of water addition. Reaction time 24 h.

Water, mM	Limonene oxide, mM	Carvone, mM	Carveol, mM	Perill aldehyde, mM	Perillyl alcohol, mM	TON
0	63	42	27	3	3	138
10	58	41	28	3	3	133
50	42	37	29	2	4	114
100	34	32	28	2	4	100

TON - product molecules per catalyst molecule

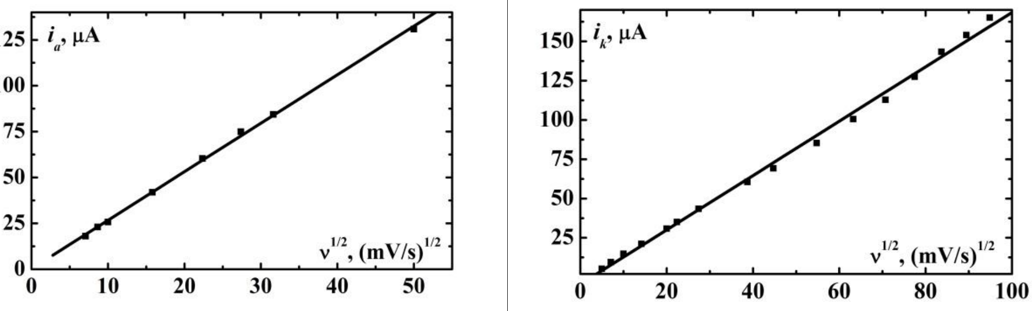

(**a**) (**b**)

Figure S1. The dependence of the current on the square root of the scan rate ($\nu^{1/2}$) registered for 5mM $[(N4Py)Fe^{II}]^{2+}$ in MeCN with 0.1 M (*t*-Bu)$_4$NClO$_4$ on a glassy carbon electrode (GCE 0.008 cm^2), for: (**a**) the anodic peak at the potential +1.05 V, (**b**) the subsequent cathodic peak.

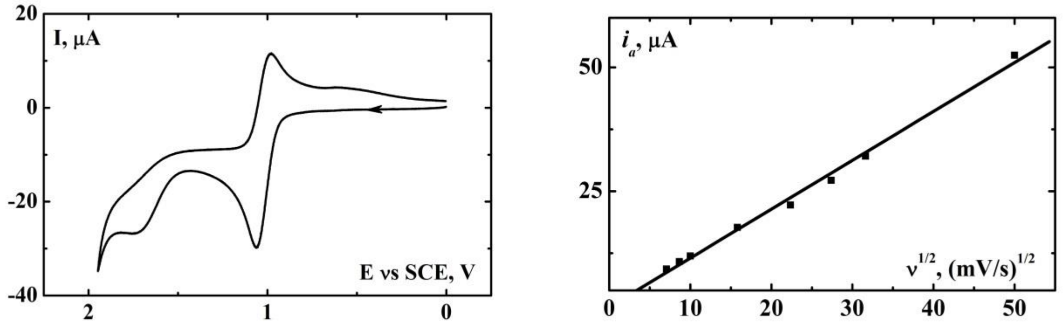

(**a**) (**b**)

Figure S2. The electrochemical behaviour of 5mM $[(N4Py)Fe^{II}]^{2+}$ in MeCN with 0.1 M (*t*-Bu)$_4$NClO$_4$, GCE (0.008 cm^2), SCE *vs.* NHE +0.242 V. (**a**) Cyclic voltammogram with anodic scan reversed after the appearance of the peak at potential +1.65 V, scan rate 0.1 V s^{-1}. (**b**) The dependence of the current on the square root of the scan rate ($\nu^{1/2}$) for the anodic peak at potential +1.65 V.

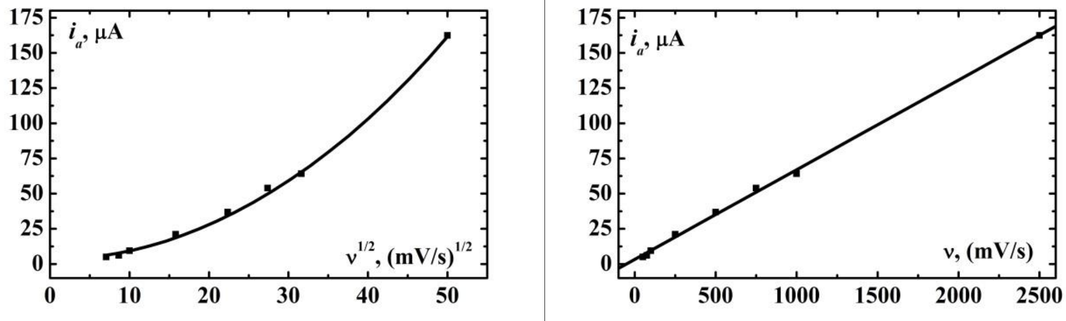

(**a**) (**b**)

Figure S3. The dependence of the current registered for the anodic peak at +1.95 V on: (**a**) the square root of the scan rate ($\nu^{1/2}$) and (**b**) the scan rate, (ν), registered for 5mM $[(N4Py)Fe^{II}]^{2+}$ in MeCN with 0.1 M (*t*-Bu)$_4$NClO$_4$ on a glassy carbon electrode (GCE 0.008 cm^2).

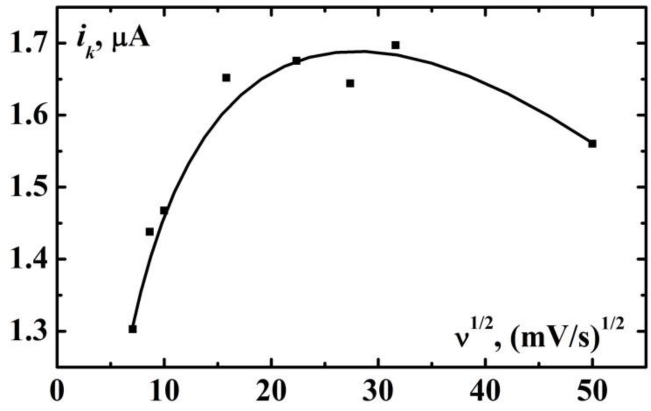

Figure S4. The dependence of the current of the cathodic peak at +1.0 V on the square root of the scan rate ($\nu^{1/2}$) registered for 5mM $[(N4Py)Fe^{II}]^{2+}$ when the scan was reversed after the appearance of the anodic peak at +1,95 V, in MeCN with 0.1 M (t-Bu)$_4$NClO$_4$ on a glassy carbon electrode (GCE 0.008 cm^2).

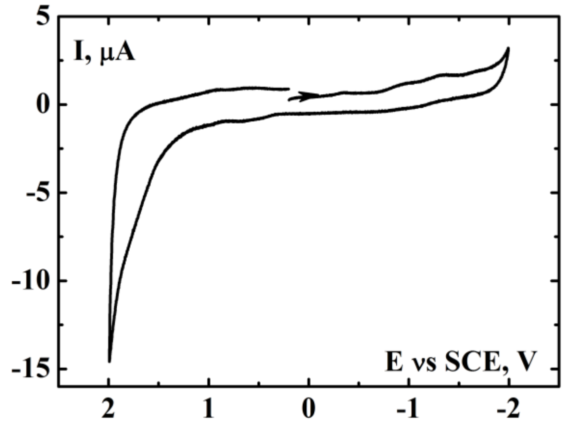

Figure S5. Cyclic voltammogram of 15 mM PhIO in MeCN with 0.1 M (t-Bu)$_4$NClO$_4$, GCE (0.008 cm^2), SCE *vs.* NHE +0.242 V.

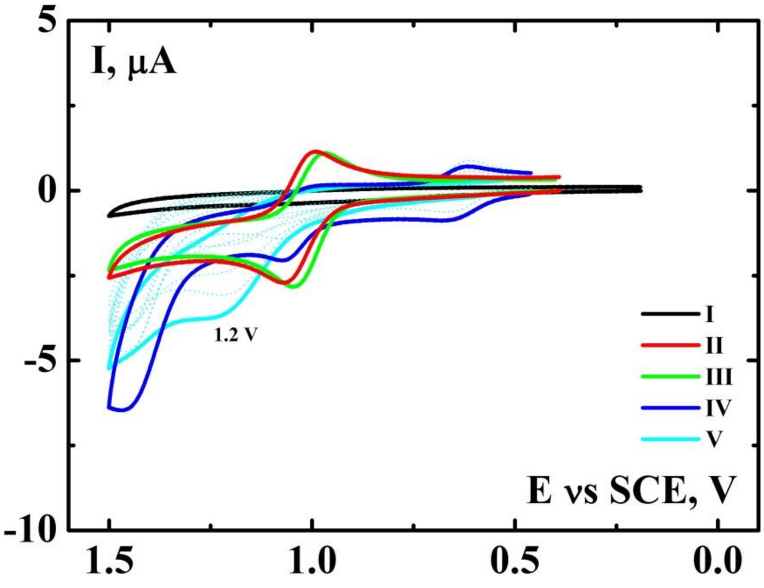

(**a**)

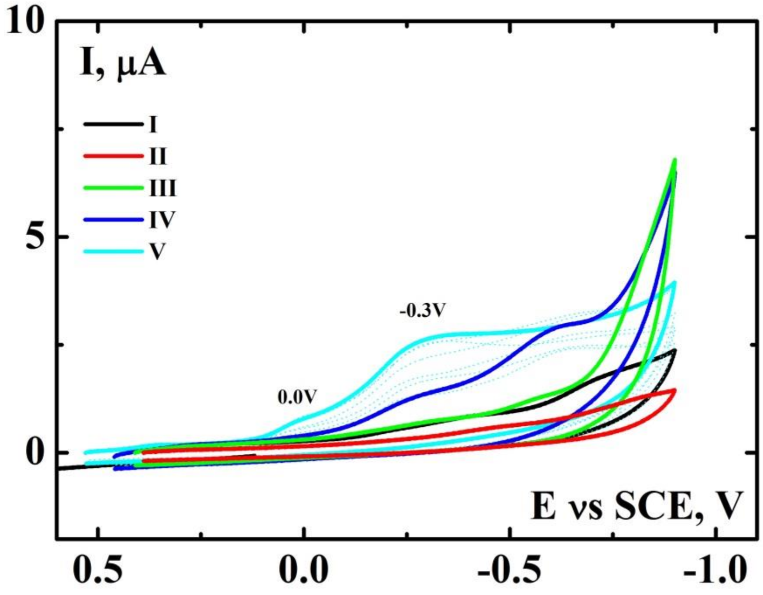

(**b**)

Figure S6. Cyclic voltammograms for 1mM $[(N4Py)Fe^{II}]^{2+}$ in MeCN with 0.1 M (t-Bu)$_4$NClO$_4$, (**a**) the anodic scan, (**b**) the cathodic scan, was recorded first.

I – basic electrolyte in Ar atmosphere, II – after addition of 1mM $[(N4Py)Fe^{II}]^{2+}$ in Ar atmosphere, III – as (II) in air atmosphere, IV – as (III) after addition 1 M cyclohexene, V – as (IV) after 5 hours. Scan rate, 0.1 V·s^{-1}, GCE (0.008 cm^2), SCE *vs.* NHE +0.242 V.

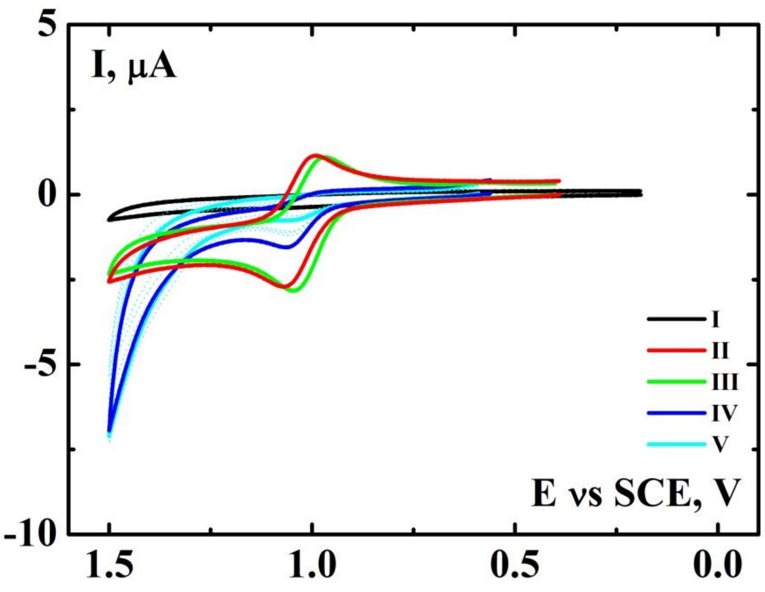

(**a**)

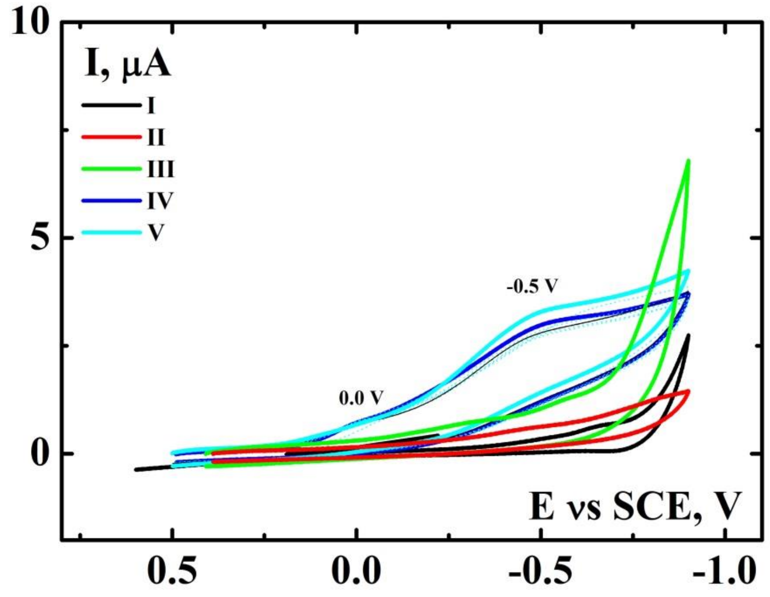

(**b**)

Figure S7. Cyclic voltammograms for 1mM $[(N4Py)Fe^{II}]^{2+}$ in MeCN with 0.1 M $(t\text{-Bu})_4NClO_4$, (**a**) the anodic scan, (**b**) the cathodic scan, was recorded first.

I – basic electrolyte in Ar atmosphere, II – after addition of 1mM $[(N4Py)Fe^{II}]^{2+}$ in Ar atmosphere, III – as (II) in air atmosphere, IV – as (III) after addition 1 M limonene, V – as (IV) after 5 hours. Scan rate, 0.1 $V{\cdot}s^{-1}$, GCE (0.008 cm^2), SCE *vs.* NHE +0.242 V.

Table S3. The energies (with and without zero point correction), enthalpies, free energies (G), and respective relative values for different catalyst molecules calculated with Def2TZVP and acetonitrile as PCM model.

**Molecules**	**Electronic Energy** [a.u.] ε_0	Sum of electronic and zero-point **Energies** [a.u.] ε_0+ZPE	Sum of electronic and thermal **Energies** [a.u.] ε_0+E_{tot}	Sum of electronic and thermal **Enthalpies** [a.u.] ε_0+H_{corr}	Sum of electronic and thermal **Free Energies** [a.u.] ε_0+G_{corr}	Relative **Electronic Energy** [kcal/mol] $\Delta(\varepsilon_0+ZPE)$	Relative **Gibbs Free Energy** [kcal/mol] ΔG
$^{1}[(N4Py)Fe^{II}]^{2+}$	–2426.961296	–2426.555019	–2426.533296	–2426.532352	–2426.605166	9.30	12.33
$^{3}[(N4Py)Fe^{II}]^{2+}$	–2426.964113	–2426.558836	–2426.536655	–2426.535711	–2426.610943	6.91	8.71
$^{5}[(N4Py)Fe^{II}]^{2+}$	–2426.973334	–2426.569843	–2426.546652	–2426.545708	–2426.624820	0	0
$^{1}[(N4Py)Fe^{IV}{=}O]^{2+}$	–2502.144940	–2501.735045	–2501.712118	–2501.711174	–2501.786248	29.55	29.87
$^{3}[(N4Py)Fe^{IV}{=}O]^{2+}$	–2502.192504	–2501.782134	–2501.759423	–2501.758478	–2501.833847	0	0
$^{5}[(N4Py)Fe^{IV}{=}O]^{2+}$	–2502.175004	–2501.766372	–2501.742741	–2501.741797	–2501.820706	9.89	8.25
$^{2}[(N4Py)Fe^{III}OH]^{2+}$	–2502.841789	–2502.420746	–2502.397471	–2502.396527	–2502.472488	1.12	4.67
$^{4}[(N4Py)Fe^{III}OH]^{2+}$	–2502.818993	–2502.400770	–2502.376273	–2502.375329	–2502.455676	13.66	15.22
$^{6}[(N4Py)Fe^{III}OH]^{2+}$	–2502.839884	–2502.422532	–2502.397488	–2502.396544	–2502.479924	0	0
$^{2}[(N4Py)Fe^{III}OOH]^{2+}$	–2578.007815	–2577.583406	–2577.558481	–2577.557537	–2577.637341	0	0
$^{4}[(N4Py)Fe^{III}OOH]^{2+}$	–2577.987598	–2577.564278	–2577.538863	–2577.537919	–2577.619846	12.00	10.98
$^{6}[(N4Py)Fe^{III}OOH]^{2+}$	–2578.000014	–2577.578317	–2577.551981	–2577.551037	–2577.636229	3.19	0.70
$^{1}[(N4Py)Fe^{III}OO\cdot]^{2+}$	–2577.35254	–2576.938850	–2576.914793	–2576.913849	–2576.991041	8.35	9.79
$^{3}[(N4Py)Fe^{III}OO\cdot]^{2+}$	–2577.364569	–2576.952162	–2576.927531	–2576.926587	–2577.006647	0	0
$^{5}[(N4Py)Fe^{III}OO\cdot]^{2+}$	–2577.352418	–2576.940662	–2576.915618	–2576.914673	–2576.995769	7.22	6.83
H atom	–0.502175248866	–0.502175249	–0.500759249	–0.499815249	–0.512829249	0.0	0.0
O_2	–150.39032754	–150.3864615	–150.3840995	–150.3831545	–150.4064175	0.0	0.0

Table S4. Reaction Gibbs free energy for the activation of complexes.

	Reactions:	Δ_rG [kcal/mol]
MeCN	$[(N4Py)Fe^{II}]^{2+}$ +0.5O_2 → $[(N4Py)Fe^{IV}{=}O]^{2+}$	–12.36
	$[(N4Py)Fe^{II}]^{2+}$ + O_2 → $[(N4Py)Fe^{III}OO]^{2+}$	6.72
	$[(N4Py)Fe^{IV}{=}O]^{2+}$ + H → $[(N4Py)Fe^{III}OH]^{2+}$	–91.86
	$[(N4Py)Fe^{III}OO]^{2+}$ + H → $[(N4Py)Fe^{III}OOH]^{2+}$	–73.96

Table S5. The energies (with and without zero point correction), enthalpies, free energies (G), and respective relative values for the singlet (**1**), triplet (**3**), and quintet (**5**) states for reaction of cyclohexene oxidation catalyzed by $[(N4Py)Fe^{IV}{=}O]^{2+}$ in PCM model.

	**Molecules**		**Electronic Energy** [a.u.] ε_0	Sum of electronic and zero-point **Energies** [a.u.] ε_0+ZPE	Sum of electronic and thermal **Energies** [a.u.] ε_0+E_{tot}	Sum of electronic and thermal **Enthalpies** [a.u.] ε_0+H_{corr}	Sum of electronic and thermal **Free Energies** [a.u.] ε_0+G_{corr}	Relative **Electronic Energy** [kcal/mol] $\Delta(\varepsilon_0+ZPE)$	Relative **Gibbs Free Energy** [kcal/mol] ΔG
**MeCN**	**Singlet**	**S**	–2736.91179188	–2736.354916	–2736.32458	–2736.323633	–2736.417462	**28.98**	**30.25**
		**TS**	–2736.90544029	–2736.350927	–2736.321653	–2736.320709	–2736.410357	**31.48**	**34.71**
		**P**	–2737.00896604	–2736.448871	–2736.41938	–2736.418436	–2736.508323	**–29.98**	**–26.76**
	**Triplet**	**S**	–2736.95835123	–2736.401096	–2736.37087	–2736.369922	–2736.465671	**0**	**0**
		**TS**	–2736.9390290	–2736.387873	–2736.358364	–2736.357420	–2736.448361	**8.30**	**10.86**
		**P**	–2736.9743620	–2736.419429	–2736.388906	–2736.387962	–2736.482233	**–11.50**	**–10.39**
	**Quintet**	**S**	–2736.94097963	–2736.385509	–2736.354346	–2736.353402	–2736.451562	**9.78**	**8.85**
		**TS**	–2736.93653845	–2736.385334	–2736.354177	–2736.353232	–2736.448848	**9.89**	**10.56**
		**P**	–2736.97598600	–2736.424122	–2736.392302	–2736.391358	–2736.489121	**–14.45**	**–14.72**

S – substrates: $[(N4Py)Fe^{IV}{=}O]^{2+}$ + H-C_6H_9, **TS** – transition state: $[(N4Py)Fe{-}{-}{-}O{-}{-}{-}H{-}C_6H_9]^{2+}$, **P** – products: $[(N4Py)Fe^{III}{-}OH]^{2+}$+$C_6H_9$

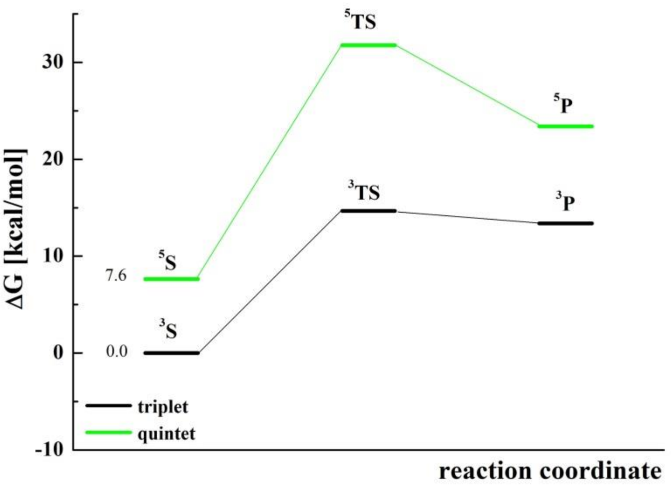

Figure S8. Relative Gibbs free energies (**Table S6**) of the triplet (**3, black**) and quintet (**5, green**) states for the reaction of 2-cyclohexen-1-ol oxidation by $[(N4Py)Fe^{IV}=O]^{2+}$ with the use of MeCN as a solvent model. For the substrates **S** (in two various spin states), the values of starting relative Gibbs free energies are given next to the corresponding levels. The symbols used: S – substrates: $[(N4Py)Fe^{IV}=O]^{2+}$ + HO-C_6H_9, TS – transition state: $[(N4Py)Fe{-}{-}{-}O{-}{-}{-}HO{-}C_6H_9]^{2+}$, P – products: $[(N4Py)Fe^{III}{-}OH]^{2+}$ + O-C_6H_9.

Table S6. The energies (with and without zero point correction), enthalpies, free energies (G), and respective relative values for the singlet (**1**), triplet (**3**), and quintet (**5**) states for reaction of 2-cyclohexen-1-ol oxidation by $[(N4Py)Fe^{IV}=O]^{2+}$ in MeCN as PCM model.

Molecules		Electronic **Energy** [a.u.] ε_0	Sum of electronic and zero-point **Energies** [a.u.] ε_0+ZPE	Sum of electronic and thermal **Energies** [a.u.] ε_0+E_{tot}	Sum of electronic and thermal **Enthalpies** [a.u.] ε_0+H_{corr}	Sum of electronic and thermal **Free Energies** [a.u.] ε_0+G_{corr}	Relative **Electronic Energy** [kcal/mol] $\Delta(\varepsilon_0$+ZPE)	Relative **Gibbs Free Energy** [kcal/mol] ΔG
**Triplet**	**S**	–2812.21408527	–2811.652184	–2811.621066	-2811.620122	-2811.716474	**0**	**0**
	**TS**	–2812.18464991	–2811.63033	–2811.599875	-2811.598931	-2811.693074	**13.71**	**14.68**
	**P**	–2812.18834658	–2811.629317	–2811.598023	-2811.597079	-2811.695145	**14.35**	**13.38**
**Quintet**	**S**	–2812.19798494	–2811.637924	–2811.605880	-2811.604935	-2811.704288	**8.95**	**7.65**
	**TS**	–2812.15140413	–2811.599094	–2811.567501	-2811.566557	-2811.665834	**33.31**	**31.78**
	**P**	–2812.16841013	–2811.610835	–2811.578657	-2811.577712	-2811.679197	**25.95**	**23.39**
**Singlet**	**S**	-2812.16271743	-2811.601970	–2811.570788	–2811.569844	–2811.664455	**31.51**	**32.64**

S – substrates: $[(N4Py)Fe^{IV}=O]^{2+}$ + HO-C_6H_9, **TS** – transition state: $[(N4Py)Fe\text{---}O\text{---}HO\text{-}C_6H_9]^{2+}$, **P** – products: $[(N4Py)Fe^{III}\text{-}OH]^{2+}$ + O-C_6H_9

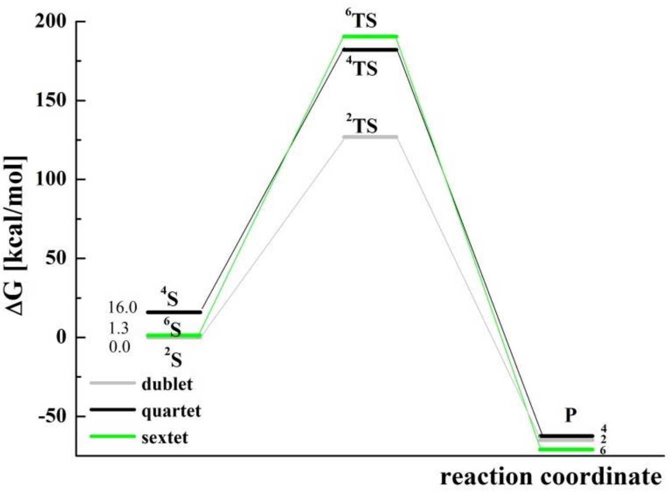

Figure S9. Relative Gibbs free energies (**Table S7**) of the doublet (**2, gray**), quartet (**4, black**), and sextet (**6, green**) states for transformations of $[(N4Py)Fe^{III}OOC_6H_9]^{2+}$ to $[(N4Py)Fe^{III}OH]^{2+}$ and ketone with the use of MeCN as a solvent model. For the substrates **S** (in three various spin states), the values of starting relative Gibbs free energies are given next to the corresponding levels. The symbols used: S – substrates: $[(N4Py)Fe^{III}OOC_6H_9]^{2+}$, TS – transition state: $[(N4Py)Fe^{III}O\text{---}OC_6H_8\text{---}H]^{2+}$, P – products: $[(N4Py)Fe^{III}OH]^{2+} + C_6H_8O$.

Table S7. The energies (with and without zero point correction), enthalpies, free energies (G), and respective relative values for the doublet (**2**), quartet (**4**), and sextet (**6**) states for the reaction of $[(N4Py)Fe^{III}OOC_6H_9]^{2+}$ (**S**) to $[(N4Py)Fe^{III}OH]^{2+}$ and ketone (**P**) in PCM model.

**Molecules**			**Electronic Energy** [a.u.] ε_0	Sum of electronic and zero-point **Energies** [a.u.] ε_0+ZPE	Sum of electronic and thermal **Energies** [a.u.] ε_0+E*tot*	Sum of electronic and thermal **Enthalpies** [a.u.] ε_0+H*corr*	Sum of electronic and thermal **Free Energies** [a.u.] ε_0+G*corr*	Relative **Electronic Energy** [kcal/mol] Δ(ε_0+ZPE)	Relative **Gibbs Free Energy** [kcal/mol] ΔG
**MeCN**	**Doublet**	**S**	−2811.57295800	−2811.021335	−2810.991401	−2810.990457	−2811.081761	**0**	**0**
		**TS**	−2811.37077126	−2810.819552	−2810.79031	−2810.789366	−2810.879464	**126.62**	**126.94**
		**P**	−2811.67056600	−2811.120549	−2811.089352	−2811.088407	−2811.185476	**−62.26**	**−65.08**
	**Quartet**	**S**	−2811.54103500	−2810.991739	−2810.960669	−2810.959725	−2811.056276	**80.83**	**15.99**
		**TS**	−2811.27022043	−2810.727827	−2810.696752	−2810.695807	−2810.791541	**184.18**	**182.12**
		**P**	−2811.66441600	−2811.115214	−2811.083485	−2811.082541	−2811.181158	**−58.91**	**−62.37**
	**Sextet**	**S**	−2811.56188700	−2811.013885	−2810.982222	−2810.981278	−2811.079681	**4.675**	**1.31**
		**TS**	−2811.25332265	−2810.712202	−2810.680441	−2810.679497	−2810.778229	**193.98**	**190.47**
		**P**	−2811.67406600	−2811.127163	−2811.094715	−2811.093771	−2811.194938	**−66.41**	**−71.02**

S – substrates: $[(N4Py)Fe^{III}OOC_6H_9]^{2+}$, **TS** – transition state: $[(N4Py)Fe^{III}O\text{---}OC_6H_8\text{---}H]^{2+}$, **P** – products: $[(N4Py)Fe^{III}OH]^{2+} + C_6H_8O$

Table S8. The energies (with and without zero point correction), enthalpies, free energies (G), and respective relative values for the singlet (**1**), triplet (**3**), and quintet (**5**) states for reaction of limonene oxidation by $[(N4Py)Fe^{IV}{=}O]^{2+}$ in PCM model.

		**Molecules**	**Electronic Energy** [a.u.] ε_0	Sum of electronic and zero-point **Energies** [a.u.] ε_0+ZPE	Sum of electronic and thermal **Energies** [a.u.] ε_0+E_{tot}	Sum of electronic and thermal **Enthalpies** [a.u.] ε_0+H_{corr}	Sum of electronic and thermal **Free Energies** [a.u.] ε_0+G_{corr}	Relative **Electronic Energy** [kcal/mol] $\Delta(\varepsilon_0$+ZPE)	Relative **Gibbs Free Energy** [kcal/mol] ΔG
**MeCN**	**Singlet**	**S**	–2893.09982685	–2892.451532	–2892.417205	–2892.416261	–2892.516227	**9.65**	**10.38**
		**TS**	–2893.04025830	–2892.398786	–2892.364447	–2892.363503	–2892.463596	**42.75**	**43.41**
		**P**	–2893.11428065	–2892.466911	–2892.431758	–2892.430814	–2892.532772	**0**	**0**
	**Triplet**	**S**	–2893.08837496	–2892.44100	–2892.406127	–2892.405182	–2892.507678	**16.26**	**15.75**
		**TS**	–2893.03789968	–2892.399143	–2892.364112	–2892.363167	–2892.466462	**42.53**	**41.61**
		**P**	–2893.07155965	–2892.428845	–2892.39296	–2892.392015	–2892.497991	**23.89**	**21.83**
	**Quintet**	**S**	–2893.10630456	–2892.460362	–2892.42479	–2892.423846	–2892.528424	**4.11**	**2.73**
		**TS**	–2893.02700674	–2892.391489	–2892.354923	–2892.353979	–2892.463922	**47.33**	**43.20**
		**P**	–2893.07337126	–2892.434600	–2892.397256	–2892.396312	–2892.507629	**20.28**	**15.78**

S – substrates: $[(N4Py)Fe^{IV}{=}O]^{2+}$ + H-$C_{10}H_{15}$, **TS** – transition state: $[(N4Py)Fe{-}{-}{-}O{-}{-}{-}H{-}C_{10}H_{15}]^{2+}$, **P** – products: $[(N4Py)Fe^{III}{-}OH]^{2+}$ + $C_{10}H_{15}$

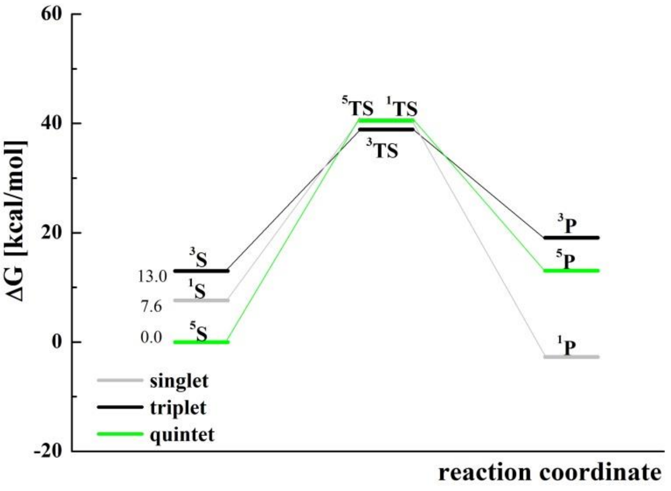

FigureS10. Relative Gibbs free energies (**Table S8**) of the singlet (**1, gray**), triplet (**3, black**), and quintet (**5, green**) states for the reaction of limonene oxidation by $[(N4Py)Fe^{IV}{=}O]^{2+}$ with the use of MeCN as a solvent model. For the substrates **S** (in three various spin states), the values of starting relative Gibbs free energies are given next to the corresponding levels. The symbols used: S – substrates: $[(N4Py)Fe^{IV}{=}O]^{2+}$ + H-$C_{10}H_{15}$, TS – transition state: $[(N4Py)Fe{-}{-}{-}O{-}{-}{-}H{-}C_{10}H_{15}]^{2+}$, P – products: $[(N4Py)Fe^{III}{-}OH]^{2+}$ + $C_{10}H_{15}$.
